# Acute Pancreatitis in Jordanian Children: A Single Center Experience

**DOI:** 10.3389/fped.2022.908472

**Published:** 2022-07-01

**Authors:** Belal Al Droubi, Eyad Altamimi

**Affiliations:** ^1^Faculty of Medicine, Jordan University of Science and Technology, Irbid, Jordan; ^2^Department of Pediatrics, Faculty of Medicine, Jordan University of Science and Technology, Irbid, Jordan

**Keywords:** pancreatitis, INSPPIRE, amylase, lipase, *CFTR* mutations, pancreas divisum (PD)

## Abstract

**Background:**

There is still much to understand and discover regarding pediatric pancreatitis. The etiology, clinical presentation, and prognosis of pancreatitis differs considerably between young children and adults. The incidence of pancreatitis has been increasing; it is no longer as rare in children as previously thought and could cause significant morbidity and mortality when severe.

**Methods:**

In this retrospective study conducted at a tertiary care hospital in Jordan, we present a cohort of children with 64 episodes of acute pancreatitis.

**Results:**

While abdominal pain was the most common presenting complaint in our cohort (97%), the classical features of radiation to the back and relief by the forward-lean position were observed in only one-third of our patients. Compared to serum amylase, serum lipase had a higher sensitivity for detecting pancreatitis (98 vs. 67%). Abdominal ultrasound is a non-invasive, widely available imaging modality; when performed, it revealed an enlarged pancreas in almost 60% of the patients. However, abdominal ultrasonography is often limited by the presence of excessive bowel gas. Anatomical abnormalities were the most common etiologies of pancreatitis (29%), followed by idiopathic pancreatitis (21%), and biliary causes (21%).

**Conclusion:**

In our cohort, serum lipase was a better diagnostic tool compared to serum amylase. Congenital biliary-pancreatic abnormalities were the most common causes of acute pancreatitis in our cohort. Almost half of these patients developed recurrent acute pancreatitis. The prevalence of pancreatic pseudocysts was 16.7%, and nearly half of them required an intervention.

## Introduction

In 2012, the INSPPIRE (International Study group of Pediatric Pancreatitis: In search for a cure) consortium published guidelines on the diagnostic criteria for pancreatitis, wherein pancreatitis was classified into acute pancreatitis (AP), acute recurrent pancreatitis (ARP), and chronic pancreatitis (CP). Based on their consensus statement, AP was defined by the presence of at least two of the following three findings: (1) abdominal pain suggestive of pancreatitis, (2) serum amylase or lipase values ≥3 times the upper limits of normal, and (3) radiological findings characteristic of pancreatic inflammation (1).

The incidence of AP is ~1 per 10,000 children per year (2, 3). Recent evidence shows that AP is becoming more frequent in the pediatric age group than previously thought (3–5). Much of our understanding of AP comes from research conducted on adult subjects (rather than on children) (6). This is concerning as there are significant differences between these two populations. The difference in etiology is a good example: in adults, the most common causes of AP are gallstones and alcohol consumption. However, in children, neither of these is a common cause; instead, the most common causes are drugs, idiopathic infections, biliary (including congenital anomalies) causes, and trauma. In addition, the distribution of the etiologies is highly diverse (6, 7).

Tomomasa et al. (8) reported a considerable discrepancy in the etiology of AP in Western children, when compared with Asian children. Their findings suggested the influence of ethnic and environmental factors. Few reports from Arabian countries have also focused on pancreatitis in children. A study from Saudi Arabia involving 50 children with AP reported that abdominal pain was the most common presenting symptom and that the most common etiologies were idiopathic (9). Interestingly, another study from Egypt involving 50 children aged above 10 years also reported abdominal pain as the most common presenting symptom and idiopathic causes as the most common etiologies (10).

In the present study, we aimed to describe the demographic profile, etiology, clinical presentation, radiological findings, and outcomes of pediatric AP. In addition, we evaluated the diagnostic criteria for AP and compared the roles of various laboratory and radiological investigations in the diagnostic process.

## Materials and Methods

### Procedure

This was a retrospective study involving children diagnosed with AP before the age of 18 years at King Abdullah University Hospital, a tertiary healthcare center in Irbid, Jordan. Electronic records of eligible patients from 2011 to 2020 were manually inserted into an Excel sheet. The data included demographic information [age, sex, and age at first recurrence (in case of recurrent pancreatitis)] and clinical data (presentation, physical findings, laboratory and radiological investigations, interventions, and outcomes). Patients older than 18 years at the time of diagnosis and those with insufficient data were excluded. Disease severity was determined based on multi-organ involvement (shock, circulatory failure, and acute respiratory distress syndrome).

### Case Identification

The diagnosis of AP was established when the patient fulfilled the INSPPIRE diagnostic criteria (1); patients who met two of the three criteria (suggestive clinical features, increased levels of serum amylase and/or lipase to values ≥3 times the upper limits of normal, and radiological findings characteristic of AP) were considered to have AP. Three of the 64 episodes of AP in this series were included based on the opinion of an expert pediatric gastroenterologist, prior to the publication of the INSPPIRE guidelines. The contribution of investigations (pancreatic enzymes and radiological findings) to the diagnosis was determined.

#### Enzyme Levels

The established cutoff values for the serum enzyme elevation were ≥303 U/L for amylase and ≥117 U/L for lipase (based on our local laboratory normal values for amylase and lipase in children).

#### Diagnostic Performance of Pancreatic Enzymes

Patients with co-ordered serum amylase and lipase tests were selected for this analysis. The accuracy of both enzyme tests in identifying AP was calculated using a 2 × 2 contingency table and tested using inferential statistical methods. The cost of utilizing both tests in our hospital was examined to suggest a cost-effective diagnostic approach.

#### Etiologies of Pancreatitis

The etiology of pancreatitis was determined retrospectively based on the available patient data.

### Ethical Approval

This study was approved by the Institutional Review Board committee (IRB #20200037) and scientific committee of the Deanship of Scientific Research of the Jordan University of Science and Technology.

### Data Analysis

Continuous variables were expressed either as mean ± standard deviation (SD) or as percentages, and where more appropriate, due to skewness of the data, we reported the median with interquartile range (IQR = 1st−3rd quartiles). Non-parametric tests were used because the data did not meet the assumptions of parametric tests. The Mann–Whitney *U*-test was used to statistically compare age and length of hospital stay (LOS) between patients who had a single attack and those who had a recurrence of AP. Fisher's exact test was performed to identify possible differences between the two groups in terms of symptoms, complications, severity, and etiology. To confirm if there was a significant difference in AP diagnostic accuracy between serum lipase and amylase, a paired sample McNemar's test was performed. All significance tests were two-tailed, with an alpha significance cutoff of *p* < 0.05. Data were imported into and analyzed using the software Statistical Package for Social Sciences (version 25.0; IBM Corporation, Armonk, NY, USA).

## Results

### Patient Characteristics

A total of 24 patients with 64 episodes of AP were identified. Patients were distributed equally by sex, and 11 (45.8%) patients had just one episode of AP. The mean age at the first AP attack was 9.8 ± 3.3 years (range = 3.45–17.7 years) (Table 1).

**Table 1:** General characteristics of patients, and the causative etiology.

	**Non-recurrence group**	**Recurrence group**	***P*** **-value^d^**	**Total (%)**
	**(*n* = 11)**	**(*n* = 13)**		**(*n* = 24)**
Age, mean years ± SD	10.3 ± 3.6	9.5 ± 3.1	0.738	9.8 ± 3.3
**Sex**
Male	6	6		12 (50)
Female	5	7		12 (50)
Length of Stay, median days (IQR)	3.5 (2.25–6)	4.5 (4–7)	0.4	4 (3–7)
**Etiology**
Anatomic^a^	2	5	0.285	7 (29)
Idiopathic	2	3	0.767	5 (21)
Biliary	4	1	0.1	5 (21)
Trauma	2	0	0.124	2 (8)
Familial/Genetic^a^	0	2	0.188	2 (8)
Drugs^b^	0	1	0.357	1 (4)
Others^c^	1	1	0.903	2 (8)

a *One patient had pancreatic divisum and genetic mutation in the same time*.

b *A child with seizure disorder was on Valproic acid*.

c *Mumps in the non-recurrence, autoimmune in the recurrence*.

d *Non-parametric Mann-Whitney U-test was used for comparing the length of stay and age across groups. Fisher's exact test was used to compare the etiology between groups, which indicates that given this sample, no significant difference in etiology was identifiable between recurrence and non-recurrence groups*.

Thirteen (54%) patients had one or more episodes of AP recurrence later; among them, the median duration between the first and second episode was 2.7 months (IQR = 44 days−11 months). The median LOS was long (4 days, IQR = 2.75–7) during the first episode and shorter (3 days, IQR = 3–5) during the recurrence; however, this difference was not statistically significant (*p* = 0.4) (Table 1).

### Etiologies of Pancreatitis

The cause of pancreatitis was identified in 19 (79%) children, while in 5 (21%) children, it was classified as idiopathic. The most commonly identified causes in our cohort were congenital biliary-pancreatic abnormalities (such as abnormal union of the pancreaticobiliary junction and pancreas divisum) (*n* = 7; 29%) and biliary causes (sludge, choledochal cyst, or gallstones) (*n* = 5; 21%). Five children had pancreas divisum, three had other abnormal findings, two had associated gallstones, and one had compound heterozygous cystic fibrosis transmembrane conductance regulator (*CFTR*) mutation. Two patients were considered to have genetic pancreatitis secondary to *CFTR* mutations (one homozygous and one compound heterozygous genotype). Autoimmune recurrent AP, suggested by very high levels of serum immunoglobulin G4 levels, was diagnosed in one patient (Table 1).

Although congenital biliary pancreatic abnormalities were common in children with recurrent AP, biliary causes were more prevalent among the non-recurrent group; however, these differences were not statistically significant (*p* = 0.285 and 0.1, respectively) Table 1.

### Clinical Features

During the 64 episodes of AP, abdominal pain was the most common presenting symptom (*n* = 62; 96%); the pain was located predominantly in the epigastric region (*n* = 53, 83%) and radiated to the back in only 21 (32%) episodes. Nausea (*n* = 45; 70%) and vomiting (*n* = 3; 52%) were less common.

Jaundice was reported in only two (3%) episodes. Nine (38%) patients manifested systemic inflammatory response syndrome (SIRS); these were considered severe episodes. However, no significant statistical difference was found regarding the incidence of SIRS between the recurrence and non-recurrence groups (*p* = 1). More details of the observed clinical features are presented in Table 2.

**Table 2:** Clinical features of acute pancreatitis in our cohort at presentation (Considering all the acute pancreatitis episodes).

**Signs & symptoms**	**Frequency**	**Total %**
Abdominal pain	62	97
Epigastric pain	53	83
Nausea	45	70
Vomiting	35	54
Abdominal tenderness	33	52
Pain radiation to back	21	33
Pain relief by leaning forward	18	28
Fever	11	17
Anorexia	9	14
Systemic inflammatory response	9	14
Diarrhea/pale stool	8	13
Abdominal distention	4	6
Jaundice	2	3
Other atypical symptoms: (LUQ pain, RUQ pain, SOB, headache, heartburn, UTI, Weight loss, constipation, HTN, hypoactivity, orthopnea, skin rash, Melena, dehydration, Loss of Consciousness)	2	Each of these occurred only once or twice

### Diagnostic Value of Pancreatic Enzymes Testing and Radiological Evaluation

Regarding the comparison between the two pancreatic enzymes and their accuracies in diagnosing AP, amylase was elevated in 42 (67%) out of 63 AP episodes, while lipase was elevated in 53 (98%) out of 54. The contingency table for lipase vs. amylase is shown in Table 3. The sensitivity of amylase to positive lipase was 42/53 (79%), while that of lipase to positive amylase was 42/42 (100%). Therefore, lipase was more often positive compared to amylase. To evaluate whether this discrepancy between the two enzymes was random, a paired sample McNemar's test was performed, and it revealed that the difference was highly statistically significant (*p* < 0.001). This indicated that lipase was more sensitive and offered greater value (in clinical setting) for the diagnosis of AP.

**Table 3:** Contingency table of lipase and amylase.

	**Amylase elevated**		
**Lipase elevated**	**No**	**Yes**	**Total**
No	1	0	1
Yes	11	42	53
Total	12	42	54*

* *Total is not equal to 64, because 10 cases were excluded from the table, as lipase was not ordered for them, while amylase was ordered for 63 of 64 AP episodes*.

Of the 64 AP episodes judged to be suitable for inclusion as episodes of AP by expert pediatric gastroenterologists, 61 (95%) met the INSPPIRE consensus criteria. During the diagnostic process, pancreatic enzymes were more often positive and therefore contributed more often to the diagnosis of AP than did radiological investigations (92 vs. 38%) (Table 4).

**Table 4:** Adherence to diagnostic criteria, and contribution of enzymes and radiology to diagnosis.

**Criteria**	**Frequency** **(% out of *n* = 64)**	**% Out of satisfied INSPPIRE**
		**(*n* = 61)**
INSPPIRE criteria met	61 (95)	–
Either enzyme positive^a^	59 (92)	96
Radiology positive^b^	24 (38)	39
Both enzymes & radiology positive	22 (34)	36

a *Either amylase or lipase elevation, Or both*.

b *Either ultrasound or CT findings suggestive of pancreatitis*.

**Figure 1:**
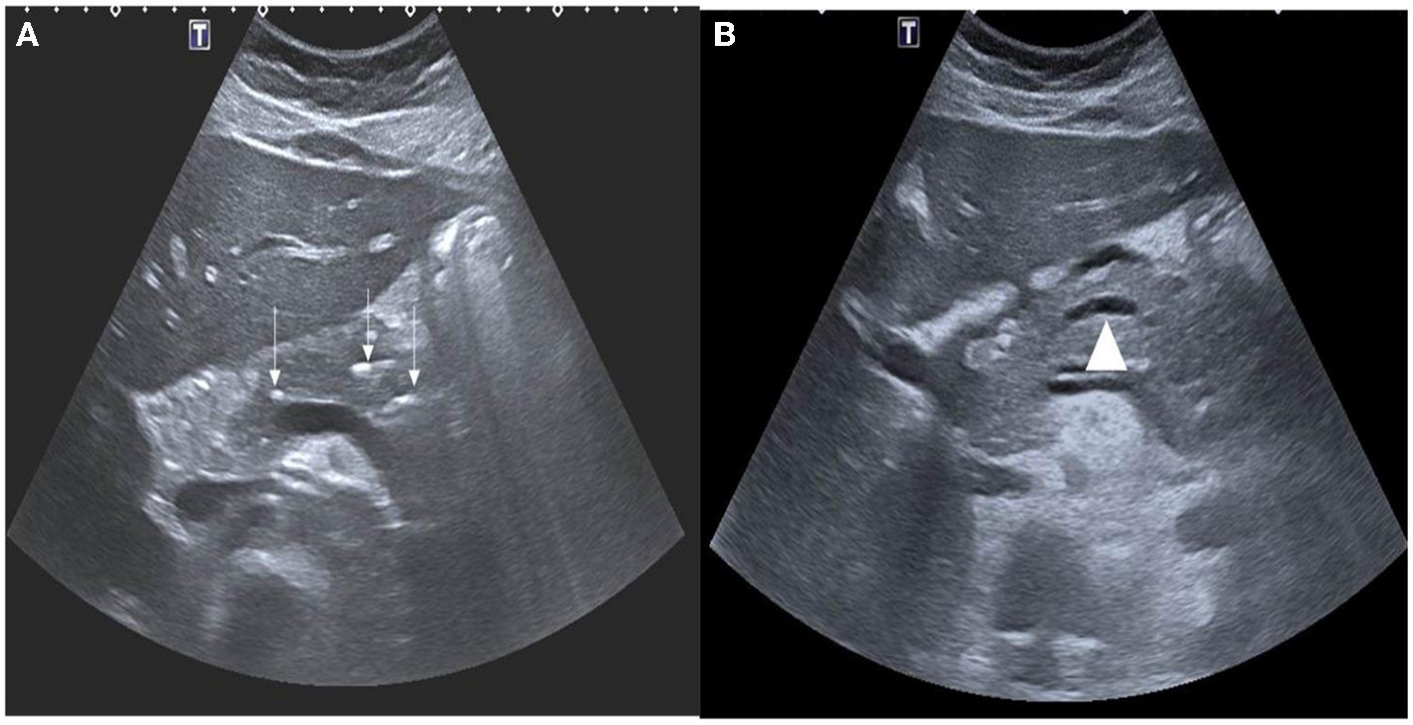
**(A)** Axial ultrasound images demonstrating multiple foci of calcifications in the pancreas (white arrows). **(B)** Notice also the dilated main pancreatic duct (arrow head).

**Figure 2:**
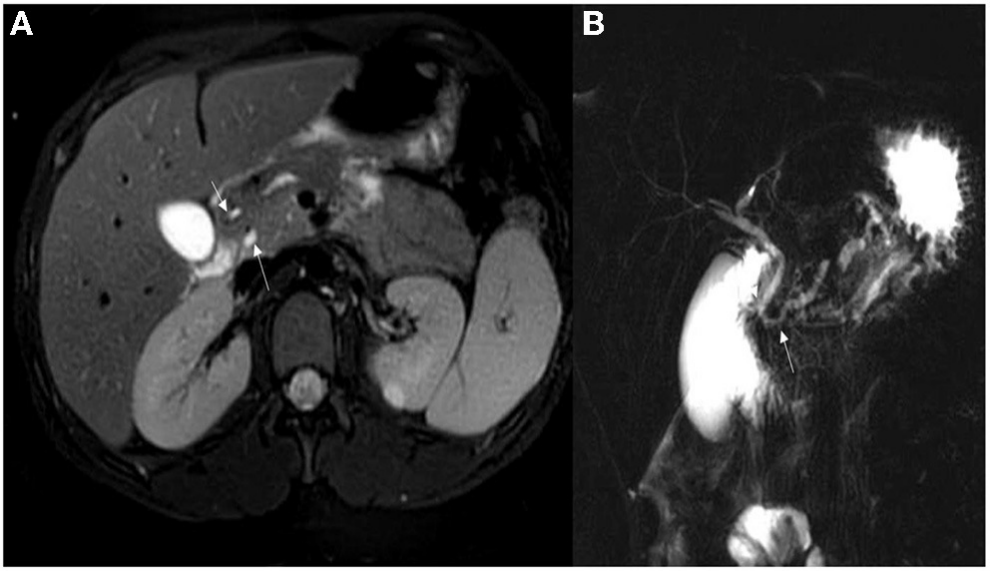
**(A)** Axial T2 WI's with fat sat demonstrating pancreatic divisum with the main pancreatic duct seen entering separately into the minor papillae (upper arrow). The lower arrow is the common bile duct seen entering into the ampulla of vater. **(B)** MRCP demonstrating pancreas divisum with the main pancreatic duct (lower arrow) open separate from the common bile duct (upper arrow).

**Figure 3:**
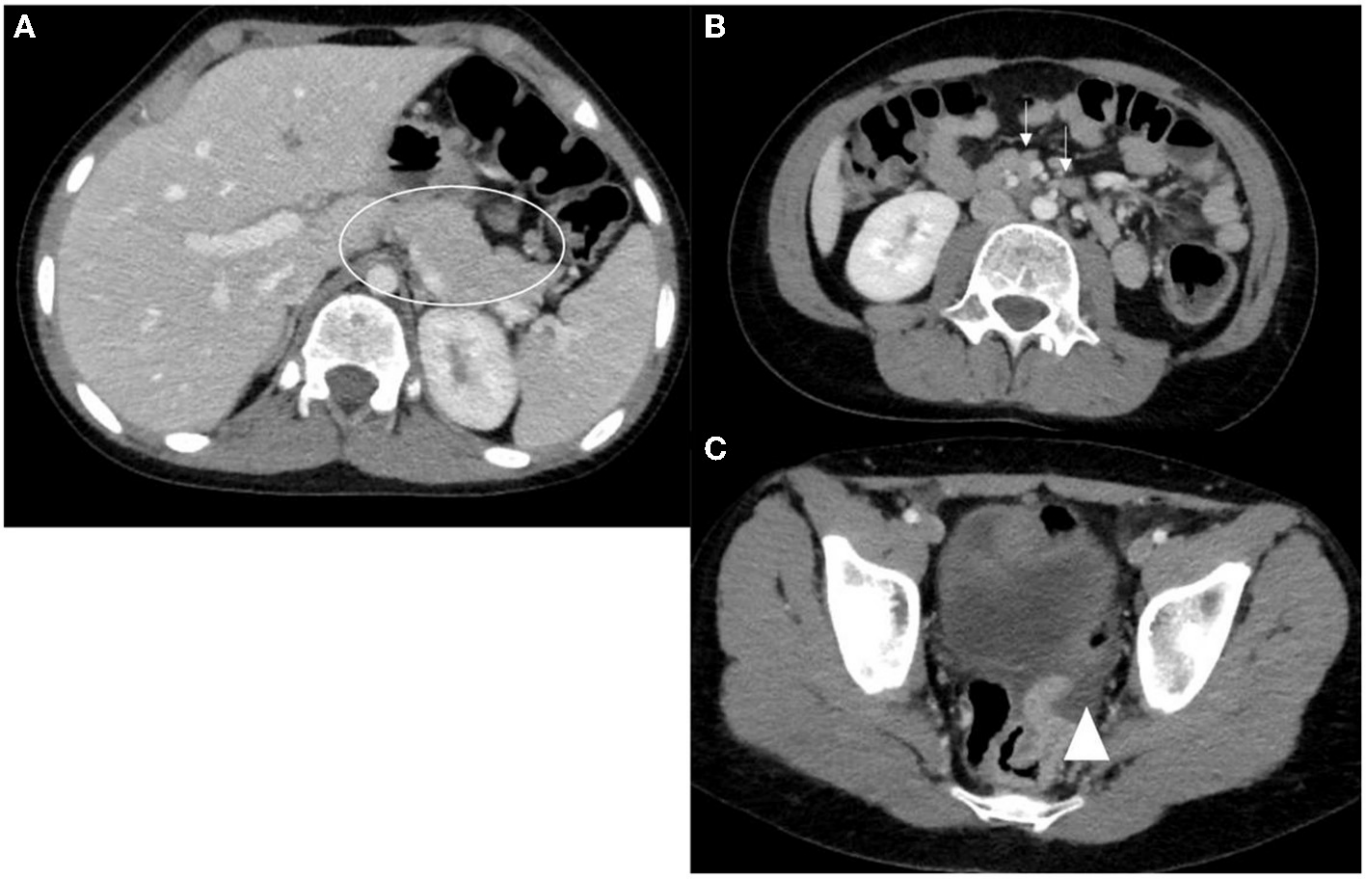
Axial CT scan images with oral and IV contrast. **(A)** Notice the bulky mildly heterogeneous pancreas (circle). **(B)** Multiple reactive peripancreatic lymph nodes (arrows). **(C)** Free fluid tracking down to the pelvis (arrow head).

Abdominal ultrasonography (US) was the most frequently used imaging modality (36 examinations). The pancreas was not visualized in 17 (47.2%) examinations because of the presence of gas in the bowels. The most common finding was a bulky and enlarged pancreas seen in 11 (58%) out of the 19 examinations where the pancreas was visualized. Magnetic resonance cholangiopancreatography (MRCP) was performed in 16 episodes; the suspicion of pancreas divisum was confirmed in three, while a normal pancreas was observed in two. In one patient, findings suggestive of cholangitis were observed, and a small choledochal cyst was visualized in one examination. One patient developed splenic vein thrombosis, which was detected on computed tomography (CT scan) (Table 5). Examples of pancreatic images and findings (US, MRI, MRCP and CT scan) presented in Figures 1–3.

**Table 5:** Radiologic findings using various imaging modalities.

**Findings:**	**US**	**CT scan**	**MRCP**
	**(36 scans)**	**(22 scans)**	**(16 scans)**
Normal	–	17	2
Non-visualized pancreas (technical)	14	–	–
Enlarged, bulky pancreas	11	–	1
Fat stranding	–	6	2
Pancreatic calcification	3	2	–
Dilated pancreatic ducts	2	6	3
Peripancreatic fluid	–	3	1
Free intrabdominal/pelvic fluid	9	11	2
Gallbladder/common bile duct stones	6	4	–
Gallbladder thickening/fluid collection	3	0	–
Dilated intrahepatic bile duct	9	3	2
Dilated extrahepatic bile duct	3	3	1
Dilated common bile duct	6	2	3
Choledochal cyst	1	2	1
Pancreatic divisum	–	–	3
Pseudocyst	1	4	–
Biliary sludge	1	–	–
Splenic vein thrombosis	–	1	–
Pleural effusion	–	4	–

### Complications of AP

The incidence rate of complications in our cohort was 25%. A peripancreatic fluid collection (pseudocyst) was observed in four (16.7%) patients among whom two required intervention (one was treated by endoscopic gastro-cystic drainage and the other by surgical drainage). One (4%) patient developed splenic vein thrombosis. It is worth noting that four patients ultimately required pancreatic replacement therapy; one of them had recurrent episodes of AP. Details of the complications observed in both groups are shown in Table 6.

**Table 6:** Pancreatitis-related complications and pathologies, stratified by patient group.

**Associated Complications**	**Non-recurrence**	**Recurrence**	**Total**	***P*** **-value**
	**(*n* = 11)**	**(*n* = 13)**	**(*n* = 24)**	
Chronic pancreatitis	0	1	1	1
Pseudocyst	1	3	4	0.596
Cholecystitis	3	1	4	0.3
Cholangitis	1	1	2	1
Pleural effusion	3	1	4	0.3
Pancreatic replacement Tx	0	4	4	0.098
Splenic vein thrombosis	1	0	1	0.458

## Discussion

Pediatric pancreatitis is a common condition. Most of our knowledge about this condition has been extrapolated from findings in adult literature. However, pediatric data are needed to estimate the true burden of this condition and appreciate the clinical presentation, etiological factors, and disease outcomes in different populations. Data from our context are scarce and to the best of our knowledge, this is the first report on pancreatitis in Jordanian children.

Recently, pancreatitis in children has gained more attention (11–13). Multiple studies have explored its etiopathogenesis and clinical presentations; meanwhile, health authorities have published guidelines for its diagnosis and management (1, 14).

The clinical presentation of AP in children is age-specific. Although abdominal pain is consistent in older children, younger children may present with more non-specific symptoms such as irritability (as a substitute for pain) and vomiting (15, 16). Compared with adults, children are less likely to manifest the classical epigastric pain which radiates to the back and is relieved by leaning forward (17). In our cohort, similar to previous literature (5, 6), these symptoms were only reported in a minority of children: only one-third of the episodes involved the classical pain. This unjustified, false labeling of presentations as “typical,” “usual,” or “classical” can contribute to poor clinical reasoning when ruling in or in this case, ruling out AP, especially by younger and inexperienced medical practitioners. Therefore, in the hope of improving patient care and medical education, it is worthwhile to reconsider this labeling or concomitantly highlight the reliability and implications of such findings during the teaching process.

Although it is well-established that serum lipase is a more sensitive and specific biomarker for diagnosing AP, no consensus has been reached regarding whether to abolish amylase testing (18). Based on the established cutoff values, lipase offered greater sensitivity than amylase for the diagnosis of AP. More precisely, it should be interpreted (while using the current INSPPIRE criteria) that lipase provides a broader and more inclusive definition of what we suspect to be AP. Several studies in literature have demonstrated the diagnostic superiority of serum lipase over amylase (18–23). In our cohort, after considering all the confirmed cases, the rate of positivity of lipase (when ordered) was higher than that of amylase (98 vs. 67%); this further supports the proposition to rely solely on lipase in the diagnosis of AP (18).

Another advantage is that lipase levels rise more rapidly and remain elevated for longer periods of time (owing to its longer half-life) when compared to amylase. This extends the diagnostic window for AP and decreases the risk of finding values below the normal limit in situations of delayed presentation or late investigations, as patients in these scenarios are more likely to be missed if we had to rely on amylase (which has a shorter half-life) (18, 22, 23). However, in our center, lipase was sometimes not ordered (10 missing results), whereas amylase was ordered for up to 63 encounters (one missing result). This was possibly due to depleted laboratory kits or the false assumption by some clinicians that amylase is of greater importance and would provide similar or even superior diagnostic findings compared to lipase and that lipase can therefore be ignored to save cost.

In low-resource centers, a two-step testing approach can be advocated, as it presents an attractive solution to the cost problem. According to Aledreesi et al. (21), this involves testing only lipase initially and instructing laboratory technicians to test for amylase if the lipase test is negative (i.e., less than a 3-fold increase).

Risk factors such as age, sex, and ethnicity also influence the incidence of AP. Inborn errors of metabolism and viral infections were seen more common in children with AP age below 10 years, while biliary causes are more commonly seen in older children, particularly females (17). Regarding ethnic and regional differences, Nydegger et al. (2) reported trauma to be the most common cause of pancreatitis in Australian children and adolescents. Studies from the United States have reported systemic diseases (4) and idiopathic (24) causes to be the most common etiologies. In a study involving Japanese children, Suzuki et al. (25) found that the leading etiologies of AP were biliary diseases. Similarly, to provide further evidence that some ethnicities are predisposed to biliary pancreatitis, a study in the United States revealed that Hispanic children diagnosed with AP were more likely to have a biliary cause than Caucasian and African American children; they noted a 3-fold and 5-fold increase in prevalence, respectively, in the Caucasian and African American children (26). These regional variations were also confirmed in a meta-analysis of 48 studies that showed that the main causes of pediatric AP were gallstones (in Asia), trauma (in Oceania), and idiopathic (in Europe and America) (27).

Although reports from the Middle East are sparse, two studies reported idiopathic etiologies as the most common causes (9, 10). A recent work in Saudi Arabia found biliary causes to be the most common, followed by idiopathic causes (21). In our cohort, congenital anomalies were the most common causes, followed by biliary and idiopathic causes. Our results are not in discordance with those in literature; our small sample size may explain the minute differences.

At least five genes have been identified to be associated with pancreatitis: serine protease 1 (*PRSS1*), serine peptidase inhibitor Kazal type 1 (*SPINK1*), *CFTR*, chymotrypsin C (*CTRC*), and calcium sensing receptor (*CASR*) (28). Genetic testing is gaining popularity in the investigation of patients with unidentified causes of pancreatitis. A genetic etiology is more associated with CP than with ARP (29). The quantity of genes related to pancreatitis cannot be precisely determined; it differs according to the tested population, genes tested, and concomitant presence of multiple genetic etiologies.

The only genetic testing available at our facility was *CFTR* gene sequencing. Patients with *CFTR* mutations may develop pancreatitis with or without cystic fibrosis. Homozygous and compound heterozygous patients with retained pancreatic sufficiency are at an increased risk (40–80 times more than the normal population) of developing CP (30). Having a complex genotype, the coexistence of other genes, and the presence of congenital abnormalities (i.e., pancreatic divisum) all increase the risk of developing pancreatitis in this population (31, 32). In our cohort, three patients had *CFTR* gene mutations (one homozygous, one compound heterozygous, and one complex heterozygous genotype); none of these patients manifested the clinical features of cystic fibrosis. The patient with a homozygous mutation had a *c.3205G*>*A* (*G1069R)* mutation, a rare variant with conflicting evidence regarding its pathogenicity. He exhibited no cystic fibrosis symptoms and had normal sweat chloride test results on different occasions. The patient had recurrent episodes of AP, whereas at his last follow-up, he had no evidence of CP. The second patient, who had a compound heterozygous genotype associated with pancreatic divisum, experienced multiple episodes of pancreatitis and ended up with pancreatic insufficiency.

According to the INSPPIRE guidelines, imaging of the pancreas represents one of the criteria for the diagnosis of pancreatitis (1) and helps in identifying the severity of the disease and its complications and in investigating the underlying cause (33). Ultrasonography is the modality of choice for evaluating children with AP. Computed tomography should be reserved for determining the severity of complicated AP or in acute traumatic settings, due to radiation concerns in children. Magnetic resonance cholangiopancreatography is used to evaluate underlying pancreaticobiliary structural abnormalities and to diagnose CP. Ultrasound in patients with pancreatitis helps to document the size of the pancreas and the presence of pancreatic duct dilatations, as well as to investigate complications (34). Ultrasonography was the most commonly used imaging modality in our cohort. Pathological findings were documented in almost 60% of the images, and our results were comparable to those of previous studies (9, 35, 36). Endoscopic ultrasonography is gaining popularity. The North American Society of Pediatric Gastroenterology, Hepatology and Nutrition recognizes endoscopic ultrasound as a safe method for investigating pancreatic disorders. However, the need for specialized training limits access to this investigation modality (37). Moreover, this modality is unavailable at our facility.

Pancreas divisum is a congenital abnormality of the pancreas, which occurs secondary to an abnormal fusion of the dorsal and ventral pancreatic ducts during fetal life (38). This malfusion causes the minor papilla to become the major drain (despite the large secretory capacity of the pancreas) and thus places a significant load on the minor duodenal papilla (39). The contribution of pancreas divisum to the etiology of pancreatitis is still an area of controversy. Fifteen percent of the general population possesses a pancreas divisum. Concomitant genetic mutations with pancreas divisum potentiate the risk of AP and explains why only a fraction of patients with pancreas divisum develop AP (40). In our cohort, five patients had pancreas divisum and only one of them had a genetic mutation (*CFTR*). Although this might suggest that pancreas divisum is an independent risk factor for AP, it could be argued that the other genetic risk factors were not tested for.

Endoscopic management of pancreas divisum is performed through endoscopic retrograde cholangiopancreatography (ERCP) and stent placement (41). This procedure is technically demanding and requires great expertise, especially in children. Only one patient in our cohort had the chance to undergo ERCP with sphincterotomy. Although he did not undergo stenting, his symptoms were relieved for some time. Unfortunately, the procedure was repeated a few months later due to a recurrence of his symptoms.

The recurrence rate after the 1st episode of AP was 54%. This is higher than the estimate 15–30% recurrence rate reported in some scholarly sources (7–9). Multiple reports have attempted to identify the risk factors for recurrence. Genetic predisposition, female sex, hyperlipidemia, severity of the first episode, and the presence of anatomic abnormalities are reportedly associated with increased risk of recurrence (29, 42).

None of the previously identified risk factors was found to be significantly associated with recurrence in our study population. We believe that this is not due to a real underlying difference in epidemiology but rather a consequence of the poor representation of these risk factors in our small cohort. The relatively small number of patients limits the possibility of creating a statistical model to predict recurrence after the first episode.

Complications of AP can be classified according to the site of development (local vs. systematic) and the timing of occurrence (early vs. late). Monitoring for complications is an important component of the management plan for patients with AP (43). The most common complication of AP is the collection of pancreatic fluid. This is the consequence of necrosis or trauma. Pancreatic pseudocysts are fibrous-walled cavities filled with pancreatic enzymes, necrotic debris, and blood. Pseudocysts formation complicates 10–23% of case of AP and up to 50% of cases of CP (44). Most of the time, this collection resolves without intervention, though surgical or endoscopic intervention may be needed (6, 45). In our cohort, the prevalence of pseudocyst formation was 16.7%, a finding which is consistent with the literature. However, 50% of the cases in our cohort required an intervention (surgical or endoscopic).

There are some limitations to this study. Its results are limited by its retrospective nature. Deficiencies in the documentation of the clinical and laboratory results could not be compensated for. In addition, the small sample size limits the generalizability of our results. The unavailability of genetic testing also affected the etiological reporting in our cohort.

In conclusion, this is the first report on AP in Jordanian children. Despite our small sample size, our patients exhibited a whole spectrum of the symptoms and signs of pancreatitis. Serum lipase performed better as a diagnostic tool compared to serum amylase. Congenital biliary-pancreatic abnormalities were the most common causes of AP. Almost half of these patients developed recurrent AP. Although the prevalence of pancreatic pseudocysts was 16.7%, half of the cases required an intervention.

## Data Availability Statement

The original contributions presented in the study are included in the article/supplementary materials, further inquiries can be directed to the corresponding author/s.

## Ethics Statement

The studies involving human participants were reviewed and approved by Institutional Review Board at Jordan University of Science and Thechnology. Written informed consent from the participants' legal guardian/next of kin was not required to participate in this study in accordance with the national legislation and the institutional requirements.

## Disclosure

Preliminary data were presented as e-posters during the Canadian Digestive Disease Week (CDDW 2022).

## Author Contributions

BA collected the data, performed statistical analysis, and drafted the first manuscript. EA conceptualized the idea, analyzed the data, and finalized the manuscript. Both authors have revised and approved the final manuscript.

## Conflict of Interest

The authors declare that the research was conducted in the absence of any commercial or financial relationships that could be construed as a potential conflict of interest.

## Publisher's Note

All claims expressed in this article are solely those of the authors and do not necessarily represent those of their affiliated organizations, or those of the publisher, the editors and the reviewers. Any product that may be evaluated in this article, or claim that may be made by its manufacturer, is not guaranteed or endorsed by the publisher.

## Abbreviations

AP: acute pancreatitis
INSPPIRE: international study group of pediatric pancreatitis: in search for a CuRE
CP: chronic pancreatitis
CFTR: cystic fibrosis transmembrane conductance regulator
MRI: magnetic resonance imaging.
